# 

**DOI:** 10.1192/bjb.2023.20

**Published:** 2024-04

**Authors:** Frederick Arthur Jack Simon

**Affiliations:** is a resident in psychiatry and psychotherapy at St Joseph Hospital Berlin Weissensee, Berlin, Germany. Email: fred.a.j.simon@gmail.com

**Figure:**
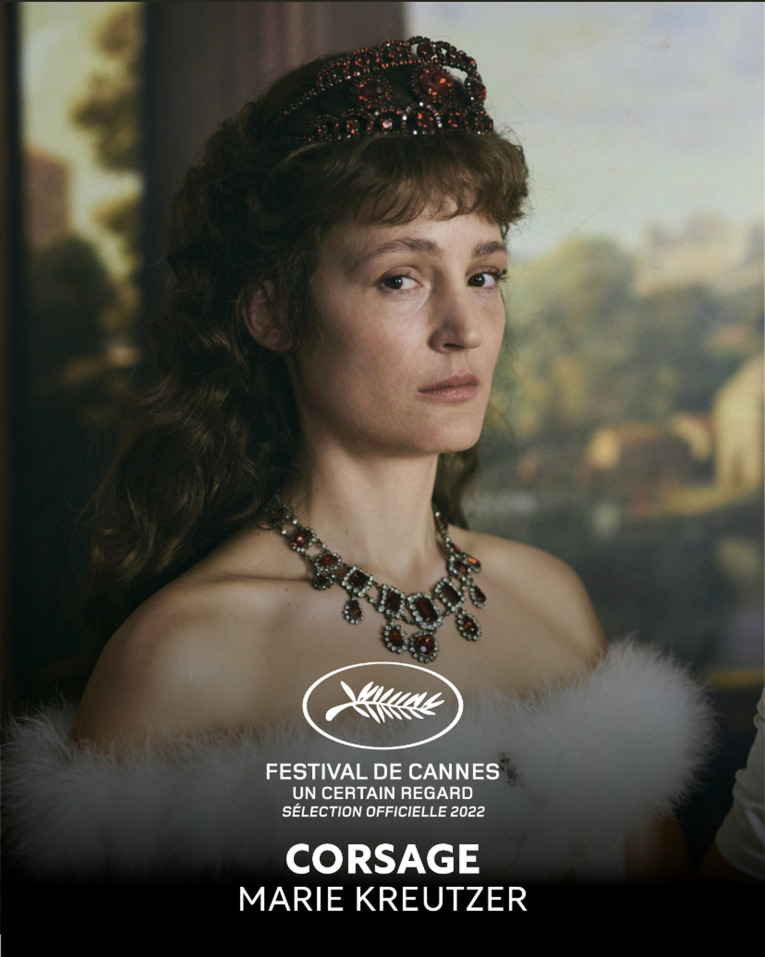

Poor Empress Elizabeth of Austria. She is miserable at the beginning of the film and dead by the end, but she is not a figure of pity. As French singer Camille prophecies in the soundtrack: ‘When she was home/She was a swan/When she was out/She was a tiger’. And in the lead role, Vicky Krieps creates a seriously tigerish tiger.

The film traces the Empress around the time of her 40th birthday, when everything ‘darkens like a cloud’ (translations from the original German are my own). Her behaviour is certainly quite unusual. She devotes time to ice baths, timing how long she can stay underwater, restricting her eating to just one slice of pre-peeled orange per meal, measuring her waist and squeezing her abdominal organs under a laced corset that gives the film its name. She sneaks into bed with her son to watch him sleep, wakes her daughter in the night to take her riding, tries to seduce her cousin, who transpires to prefer stable-boys, attempts suicide multiple times, is bullied by her husband, causes the death of a horse and eventually kills herself after training a doppelgänger to be able to continue her reign. It sounds absurd, but it is an extraordinary film.

Kreutzer does not allow herself to be confined by the literal truth of history: anachronisms abound and alternative endings appear. But reality is not the aim, nor the achievement. The film is a close study of psychic turmoil and the lasting conflicts between dependence and autonomy, submission and control, desire for care and autarchy.

Moments of psychodynamism are woven into the script. Her closest lady-in-waiting writes that the Empress is ‘a book for me. On every page stands a puzzle. Within her, everything is like a disordered museum. […] She lives in another world and goes through it on her own, and her path is only ever wide enough for one person’. The Empress's son later admonishes his mother for her indiscreet behaviour: ‘with my deepest admiration, Mama, it is you who is the child, when you give in to every impulse and every whim, without thinking about your role’. She exclaims her fragility in her own words, saying ‘people fear transience. One breath and their life is over. They would do anything to hold on to some of it […] I can't hold on to anything except myself’.

While having her portrait painted the Empress declares ‘the main thing is that we leave behind a pretty picture’. Marie Kreutzer and Vicky Krieps have done much more than create a pretty picture. Their volatile Empress is not a historical victim. She is a queen who learns to determine her own existence.

